# Online Friction Measurement and Wear-Life Determination for Textile Needle Hooks Based on Closed-Loop Tension Control and the Capstan Model

**DOI:** 10.3390/s26103011

**Published:** 2026-05-10

**Authors:** Yongkang Chen, Yang Zeng, Wang Xu, Hong Gan, Mi Xiao, Jianyu Zhu, Yuqin Wu, Pei Wang, Shunqi Mei, Lianqing Yu

**Affiliations:** 1 Hubei Key Laboratory of Digital Textile Equipment, Wuhan Textile University, Wuhan 430200, China; 2 School of Mechanical Engineering and Automation, Wuhan Textile University, Wuhan 430200, China

**Keywords:** textile needle hook, capstan model, equivalent friction coefficient, closed-loop tension control, signal-processing pipeline, wear-life determination

## Abstract

**Highlights:**

- An integrated methodology for online measurement of friction performance and wear-life determination of textile needle hooks is proposed.
- The equivalent friction coefficient is derived from real-time tensions measured on both sides of the yarn–needle wrap interface, under multi-constraint validity screening and processing to ensure estimation reliability.
- Pre-filtering and post-filtered differentiation are employed to suppress mechanical disturbances and measurement noise, thereby significantly enhancing the smoothness and robustness of the friction coefficient curve.
- A wear-life criterion based on the “relative threshold + persistence time” approach reduces false triggers and enables more robust, operationally meaningful lifetime determination.

**Abstract:**

This paper proposes an online wear monitoring and lifetime assessment method for textile needle hooks, based on yarn tension sensing, closed-loop tension control, and the capstan model. The yarn tensions on both sides of the yarn–needle wrap interface are measured in real time and used to estimate an equivalent friction coefficient, which serves as the monitoring index for wear evolution. Closed-loop average-tension control was employed to reduce variability in operating conditions and enhance the consistency of friction coefficient estimation. To improve robustness, the signal-processing pipeline includes tension-floor gating, ratio clipping, missing-data handling, outlier rejection, pre-filtering, and post-filtered differentiation. Wear-life determination is achieved through a baseline-referenced criterion that combines a relative threshold with persistence time, defining the life endpoint as the earliest sustained deviation from the steady-stage baseline, rather than isolated spikes. Experiments conducted on needle hooks of different quality grades demonstrate that the proposed method yields stable yarn-tension measurements, enables clear discrimination among wear states, and produces wear-life assessments consistent with offline microscopy observations. The aforementioned method is computationally lightweight and suitable for practical online wear monitoring, thereby enabling data-driven timing of needle replacement in looms.

## 1. Introduction

In textile manufacturing, needle hooks are continuously subjected to yarn contact, cyclic motion, and frictional loading, making them typical wear-sensitive components during long-term service. Previous studies have shown that the wear and failure of knitting needles are closely related to the hook region, operating parameters, material characteristics, and structural design. A recent review summarized that fatigue wear is the main failure mode of circular weft knitting needles and that wear is mainly concentrated at the needle-hook area [1]. The dynamic behavior of circular knitting needles has also been investigated under different yarn, machine, and process parameters, showing that machine speed, yarn tension, fiber type, and yarn input tension can affect needle displacement during loop formation [2]. From the material and structural viewpoint, the wear resistance of knitting machinery needles has been related to chemical composition, microstructure, mechanical properties, surface treatment, carbide morphology, and hook-region stress/strain distribution [3]. These studies indicate that needle-hook wear is not a purely local surface phenomenon, but a coupled result of yarn–needle interaction, operating conditions, material state, and structural response.

With the development of intelligent textile manufacturing and predictive maintenance, data-driven monitoring has become increasingly important for improving equipment reliability and maintenance decision-making. Intelligent automation has been introduced into knitting manufacturing to improve design and production efficiency [4], and machine-learning/IoT-based predictive maintenance has shown value for industrial maintenance support [5]. These studies provide useful background for intelligent production and maintenance-oriented decision-making. However, for textile needle hooks, the key difficulty remains how to construct a reliable online indicator that can reflect wear evolution and support life-level evaluation under actual operating conditions.

Existing studies on needle-hook failure and wear have mainly relied on offline or post-test evidence. Hook failure of latch needles has been analyzed from the viewpoint of dynamic loading and fatigue-related breakage [6]. SEM stereoscopy has been used to explain the wear behavior inside latch needle hooks under different yarn and machine parameters [7]. Fatigue-life estimation of circular knitting needles has also been studied by combining finite-element analysis with fatigue-life calculation [8]. These works provide important understanding of needle-hook failure mechanisms, wear morphology, and fatigue life. Nevertheless, they mainly focus on failure analysis, SEM-based characterization, numerical simulation, or post-test evaluation. They do not establish an online measurement-to-life evaluation chain that can convert short-duration yarn–hook measurements into a repeatable wear-life output.

Online monitoring and friction modeling provide possible technical foundations for this problem. Triboelectric signals and frictional vibration signals have been used to monitor lubrication or wear states in general tribological systems [9,10]. Yarn-to-yarn friction behavior has been studied to reveal how yarn structure affects frictional response during textile processing [11]. In addition, inter-yarn friction models, statistical friction analysis, and nonlinear friction models considering relative velocity have been developed to describe textile friction behavior under different conditions [12,13,14]. These studies demonstrate that measurable signals and friction-related models can reflect contact-state or wear-state changes. However, they mainly focus on generic friction pairs, yarn-to-yarn friction, or textile friction modeling, and the potential relationship between friction evolution at the yarn–needle-hook interface and needle-hook wear-life behavior has not been specifically addressed.

For yarn wrap-around contact, the capstan relation provides a physically interpretable basis for connecting two-side yarn tension with interfacial friction. Singh studied the planar equilibrium of an elastic rod wrapped around a circular capstan and discussed contact states under capstan-type wrap-around contact [15]. This theoretical relation makes it possible to estimate an equivalent friction coefficient from the two measured yarn tensions around the needle-hook contact. However, the capstan relation alone cannot solve the practical problems of online measurement reliability, operating-condition drift, abnormal tension samples, baseline extraction, and wear-life endpoint determination. Therefore, the remaining problem for textile needle hooks is not simply whether a friction-related signal can be measured, but how this signal can be converted into a robust online life indicator.

This problem is particularly challenging because the needle hook is small, the local yarn–hook contact region is difficult to observe directly during operation, and the measured tension signals are strongly coupled with yarn properties, yarn-path fluctuation, lubrication state, rotational-speed variation, installation error, and environmental disturbances. In addition, actual service life is affected by yarn type, machine condition, process parameters, and maintenance environment, making a universal month-level life value difficult to define. Therefore, a physically interpretable online indicator, a stable measurement-control condition, and a repeatable engineering criterion are required for needle-hook wear monitoring and life-level evaluation under controlled operating conditions.

**Figure 1:**
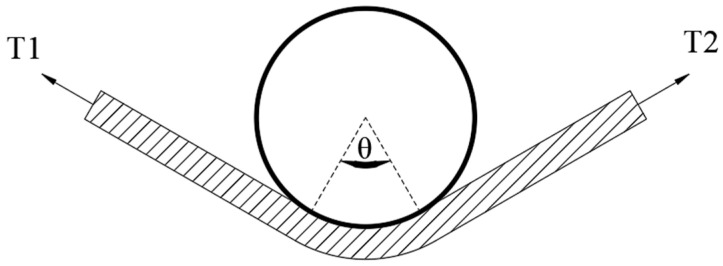
Schematic illustration of yarn wrap-around contact based on the capstan model.

For yarn wrap-around contact, the classical capstan model provides the theoretical relation between the two-side yarn tensions and the interfacial frictional state. The relation can be expressed as:

(1) $$T2=T1e^{\mu \theta }$$

where μ denotes the friction coefficient at the yarn–needle hook interface, θ represents the yarn wrap angle, and T1 and T2 are the yarn tensions on the slack and tight sides, respectively, as illustrated in Figure 1. The equivalent friction coefficient μ can be expressed as:

(2) $$\mu =\frac{1}{\theta }ln(\frac{T2}{T1})$$

It should be emphasized that the μ obtained in this study is not treated as a universal material-specific coefficient of friction. Instead, it is defined as an equivalent friction coefficient under a fixed yarn path, wrap angle, yarn condition, and operating setting. Under these controlled conditions, the evolution of μ reflects changes in yarn–hook tension-transfer behavior and is therefore used as an online indicator of wear evolution.

Although the capstan model and threshold-based decision rules are established techniques, their direct use is insufficient for practical needle-hook wear-life evaluation. In this study, two-side tension sensing, closed-loop average-tension control, data-quality gating, equivalent-friction estimation, and baseline-referenced persistent-exceedance judgment are integrated into a unified online evaluation framework.

The main contributions of this study are summarized as follows.

1. An equivalent-friction indicator is constructed from two-side yarn-tension measurements using the capstan relation.
2. Closed-loop average-tension control and data-quality screening are combined to improve the stability and reliability of online friction estimation.
3. A baseline-referenced persistent-exceedance criterion is used to define the online wear-life endpoint, and its feasibility is evaluated through repeatability, quality-grade discrimination, and consistency with offline wear characterization.

The long-term objective of this research is to support practical replacement-interval estimation for textile needle hooks. The present study focuses on the feasibility of the proposed online engineering endpoint under controlled conditions, while direct month-level calibration with field service life requires larger datasets and long-term maintenance records.

## 2. Materials and Methods

### 2.1. Method Framework

Online evaluation of needle-hook wear requires a stable and traceable chain from measured signals to maintenance-oriented decisions. In this study, the slack-side tension $T_{1}$ and tight-side tension $T_{2}$ around the yarn–needle-hook wrap contact are used as the basic observables. Under a fixed wrap-contact configuration, these two tensions are converted into an equivalent friction coefficient μ based on the capstan relation. The long-term evolution of μ is then used to determine an online engineering wear-life endpoint $t_{life}$.

The overall framework consists of four sequential steps:

1. Two-side yarn-tension acquisition under closed-loop average-tension control;
2. Sample validity screening and anomaly rejection to ensure that only physically reliable data enter the ratio-logarithm calculation;
3. Online computation of the equivalent friction coefficient under fixed sign and geometry conventions;
4. Baseline extraction and persistent-exceedance judgment for wear-life endpoint determination.

In this framework, μ is interpreted as an equivalent indicator of tension-transfer behavior rather than a universal material coefficient of friction. Therefore, comparability is ensured within controlled operating conditions, where the wrap angle θ, yarn condition, contact path, and tension setting are kept consistent. The role of closed-loop tension control is to reduce operating-condition drift, while the role of data-quality control is to prevent invalid or abnormal samples from contaminating friction estimation, baseline extraction, and life judgment.

### 2.2. Online Computation of the Equivalent Friction Coefficient

To ensure reproducibility and cross-sample comparability, unified conventions are adopted for the two measured tensions and for the online computation of the equivalent friction coefficient. At each sampling instant k, the system acquires two tension-sensor readings. After installation calibration, a fixed mapping is used to assign them to the slack-side tension T1[k] and the tight-side tension T2[k]. This fixed convention avoids ambiguity in interpretation and preserves the physical meaning of the two-side tension transfer during the whole life test. If channel swapping occurs, the assignment of $T_{1}$ and $T_{2}$ should be checked and fixed before the test based on the actual installation positions.

Based on the capstan relation introduced in the Introduction, the equivalent friction coefficient μ[k] is computed sample by sample as follows:

(3) $$\mu [k]=\frac{1}{\theta }ln(\frac{T2[k]}{T1[k]})$$

where θ is the yarn wrap angle in radians. In this study, θ is determined by the yarn path geometry and calibrated before testing. It is then treated as constant within a long-duration life test so that the computed μ[k] remains comparable over time. For convenience, the tension ratio R[k] may also be written as:

(4) $$R[k]=\frac{T2[k]}{T1[k]}$$

From Equation (4), μ[k] can be expressed as follows:

(5) $$\mu [k]=\frac{1}{\theta }ln(R[k])$$

This computation provides a direct online link from the measured two-side tensions to the equivalent friction coefficient used for wear evaluation.

In subsequent processing, μ[k] is admitted into baseline extraction and life-criterion computation only when the corresponding sample satisfies the validity constraints described in Section 2.3. This requirement is necessary because the ratio-logarithm operation can amplify low-tension errors, abnormal ratios, and asynchronous disturbances.

Besides μ, the tension difference ΔT and the tension ratio R are recorded as auxiliary diagnostic variables. However, wear-life determination uses μ as the main input because it directly characterizes the two-side tension-transfer state under the fixed wrap-contact condition. The validity constraints and anomaly-handling rules used before admitting samples into baseline extraction and wear-life determination are described in the following section.

### 2.3. Signal Processing, Data-Quality Control, and Outlier Rejection

#### 2.3.1. Validity Constraints and Sample Screening

Because the calculation of μ involves a ratio-logarithm operation, low-tension samples, abnormal tension ratios, missing packets, and asynchronous disturbances may be amplified and may contaminate baseline extraction and wear-life judgment. Therefore, explicit validity constraints are applied before friction estimation. Only samples satisfying these constraints are assigned q[k]=1 and admitted into μ[k] computation, baseline statistics, and persistent-exceedance counting. Invalid samples are assigned q[k]=0 and are excluded from all life-decision calculations.

1. Tension gating. A lower tension bound $T_{min}$ is defined to prevent unstable ratio-logarithm calculation under near-zero or low-tension conditions. If $T_{1}[k]<T_{min}$ or $T_{2}[k]<T_{min}$, the sample is marked as invalid with q[k]=0, and μ[k] is treated as undefined. Only samples with q[k]=1 are allowed to enter subsequent baseline extraction and wear-life criterion computation. In this experiment, the lower bound is set as:
  (6) $$T_{min}=0.05\bar{T},$$
  where $\bar{T}$ is the average yarn tension regulated by proportional-integral-derivative (PID) controller, ideally $\bar{T}=(T1+T2)/2$.
2. Ratio clipping and validity checking. For samples passing tension gating, check bounds $R[k]\in [R_{min},R_{max}]$. If the ratio violates the bounds, mark the sample invalid (q[k]=0) to prevent nonlinear blow-ups in the log derivation. $R_{min}$ is suggested as 1.00 or 1.01 to avoid non-physical μ[k]<0. For visualization, a clipped ratio may be displayed, but it must not be counted as valid. $R_{max}$ can be set by:
  (7) $$R_{max}=e^{\mu_{max}\theta }.$$

In this experiment, $R_{min}=1.01$, θ=105°, and $R_{max}=4.2$ to avoid saturation within expected $\mu_{max}$ and θ. Clipping (ratio-violation) events are counted only as a diagnostic for data quality (e.g., the window-level valid-sample ratio $N_{eff}/N_{ss}$ in Section 2.4.2) and are excluded (q[k]=0) from μ computation, baseline statistics, and wear-life counting.

3. Missing-data and timestamp-discontinuity handling. When packets are missing or timestamps are discontinuous, the affected samples are marked as invalid with q[k]=0 and are excluded from friction-coefficient estimation, baseline statistics, and wear-life criterion computation. Let m denote the number of consecutive missing samples. If m is small, interpolation may be used only for visualization continuity. If the interruption duration remains shorter than 1 s, the last valid value may be displayed as a held value. Once the missing duration exceeds 1 s, a safe stop is triggered. In all cases, interpolated or held values are not treated as valid samples for life-decision calculations.

**Figure 2:**
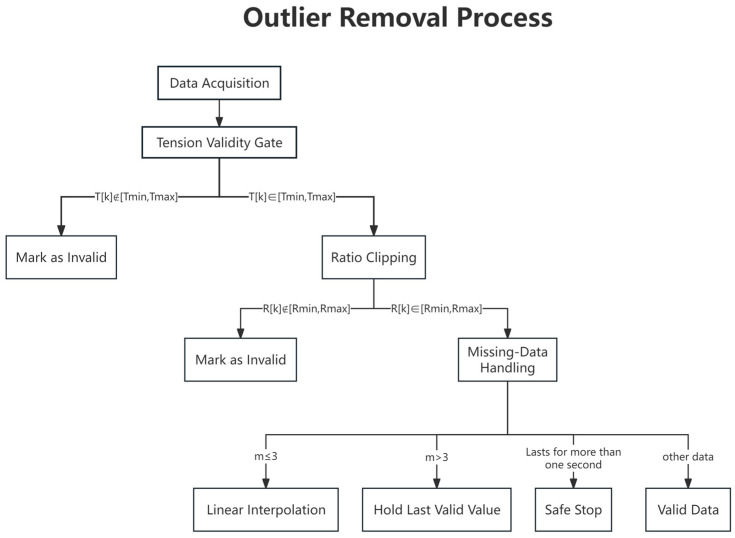
Outlier rejection and data-quality control workflow.

The above validity constraints and rejection rules define how raw tension samples are screened before they are admitted into friction-coefficient estimation, baseline extraction, and wear-life decision. Their logical sequence, including minimum-tension gating, ratio-bound checking, and missing-data exclusion, is summarized in Figure 2.

#### 2.3.2. Noise Suppression and Filtering Strategy

After invalid samples are excluded by the quality-gating rules described above, filtering is introduced mainly to improve closed-loop stability and to reduce the influence of measurement noise on the control channel. Since the equivalent friction coefficient is obtained through a ratio-logarithm operation, random fluctuation, packet jitter, and high-frequency tension ripple may still increase the variance of μ[k], even when the samples are valid. However, filtering is not used to convert invalid data into valid data. Samples marked with q[k]=0 remain excluded from friction-coefficient estimation, baseline extraction, and life-criterion counting.

In the closed-loop control channel, the average yarn tension $\bar{T}=(T_{1}+T_{2})/2$ is used as the feedback variable. Previous studies on textile yarn tension control have shown that feedback filtering, signal processing, and adaptive or cascade control strategies are effective for reducing measurement noise and improving dynamic tension regulation [16,17]. Following this established control logic, a first-order pre-filter is applied to the feedback signal before it enters the PID controller, so that the controller responds mainly to slow tension drift rather than high-frequency measurement ripple. When the derivative term is enabled, the derivative channel is also low-pass filtered to avoid direct amplification of high-frequency noise. In addition, a small error deadband and a speed-command slew-rate limit are used to reduce actuator dithering and mechanical excitation. Since these measures are mature control-side noise-suppression procedures, only their functional roles and parameter settings are reported here, rather than deriving their standard discrete forms.

**Figure 3:**
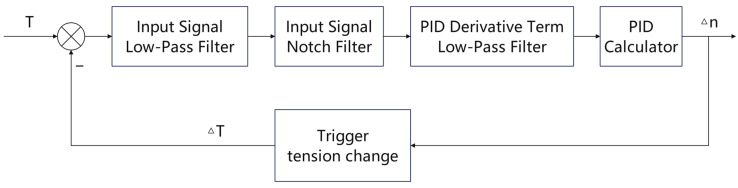
Block diagram of closed-loop tension control.

The complete signal-conditioning and closed-loop control structure is shown in Figure 3. In this structure, validity gating determines whether a sample is allowed to enter life-decision statistics, while pre-filtering, derivative filtering, deadband processing, and slew-rate limiting improve the stability of the tension-control loop.

In this experiment, the feedback pre-filter cutoff frequency was set to $f_{c}=2\;Hz$, the sampling period was $T_{s}=0.02\;s$, the control deadband was set to $e_{d}=0.02\;N$, and the maximum step-to-step speed-command increment was limited to 16 rpm, corresponding to 800 rpm/s at 50 Hz. When derivative action was enabled, the derivative-channel low-pass cutoff frequency was set to $f_{d}=5\;Hz$. These parameters were selected to suppress high-frequency ripple while maintaining sufficient response to slow tension drift.

In addition to the standard low-pass filtering described above, a speed-related adaptive notch filter is introduced to suppress dominant periodic tension ripple caused by eccentricity, guide-wheel runout, assembly clearance, or transmission disturbance. This part is important because periodic mechanical ripple may be tracked by the controller and converted into unnecessary speed modulation, which further excites the mechanical structure and increases tension fluctuation. Simply lowering the low-pass cutoff frequency can suppress this ripple, but it also introduces additional phase delay and weakens the response to slow tension drift. Therefore, when a dominant periodic component is observed, a narrowband notch filter with a speed-dependent center frequency is used.

The notch center frequency is estimated from the motor speed as:

(8) $$f_{0}=\frac{n}{60}\cdot i\cdot h$$

where $f_{0}$ is the notch center frequency, n is the motor speed in rpm, i is the transmission ratio from the motor to the disturbance-carrying rotating element, and h is the harmonic order of the dominant disturbance. In this experiment, i=1 and h=1, so:

(9) $$f_{0}=\frac{n}{60}$$

A key point of the adaptive notch design is that the rejected absolute bandwidth should remain approximately constant under different speeds. Since the quality factor Q is defined as the ratio between the center frequency and the rejected bandwidth, a higher center frequency requires a larger Q value to maintain a similar absolute bandwidth. Therefore, the target quality factor is defined as:

(10) $$Q^{*}=\frac{f_{0}}{B_{w}}$$

where $B_{w}$ is the desired rejected absolute bandwidth. To avoid an excessively wide or excessively sharp notch, the target quality factor is constrained within a prescribed range:

(11) $$Q_{tar}=min(max(Q^{*},Q_{min}),Q_{max})$$

In this experiment, $B_{w}=0.8\;Hz$, $Q_{min}=2$, and $Q_{max}=12$. When the motor speed is below the activation threshold $n_{min}=30\;rpm$, the notch filter is bypassed to avoid unnecessary coefficient updates in the low-speed region.

To prevent coefficient jitter caused by small speed fluctuations, both the center frequency and the quality factor are updated through first-order smoothing:

(12) $$f_{0}[k]=f_{0}[k-1]+\alpha_{f}(f_{0,tar}[k]-f_{0}[k-1])$$

(13) $$Q[k]=Q[k-1]+\alpha_{Q}(Q_{tar}[k]-Q[k-1])$$

where $\alpha_{f}$ and $\alpha_{Q}$ are smoothing factors for the center frequency and the quality factor, respectively. In this experiment, $\alpha_{f}=\alpha_{Q}=0.2$. The notch coefficients are recomputed only when the difference between the smoothed parameters and the last applied parameters exceeds the update thresholds:

(14) $$|f_{0}[k]-f_{0,last}|>\Delta f_{0,th}\;or\;|Q[k]-Q_{last}|>\Delta Q_{th}$$

where $f_{0,last}$ and $Q_{last}$ denote the last applied center frequency and quality factor, respectively. In this experiment, $\Delta f_{0,th}=0.2\;Hz$ and $\Delta Q_{th}=0.5$. This smoothed and thresholded update strategy reduces frequent coefficient recomputation and improves numerical stability during long-duration wear tests.

It should be noted that the filtering operations described in this subsection are mainly applied to the control and signal-conditioning channels to improve closed-loop stability. The life-decision logic still follows the quality flag q[k], the steady baseline $\mu_{ss}$, and the persistent-exceedance rule described in Section 2.4. Therefore, short-term filtering delay or amplitude attenuation is not used as a direct failure trigger. Instead, the wear-life endpoint is determined only when valid samples show a sustained exceedance relative to the steady baseline. This design reduces false triggering caused by transient noise, periodic ripple, or sporadic invalid samples.

### 2.4. Wear-Life Determination Criterion

#### 2.4.1. Staged Wear Assumption and Decision Target

Under wrap-around yarn–hook contact, the measured tension-transfer behavior changes progressively with the surface condition of the needle hook. During the early running-in stage, micro-asperities on the contact surface and the local yarn–hook contact state may undergo rapid rearrangement, and the equivalent friction coefficient μ may fluctuate or change slowly. During the steady-wear stage, if the wrap angle, yarn path, yarn condition, and tension setpoint remain stable, μ usually fluctuates within a limited range around a steady baseline. When the hook surface becomes blunt, roughened, or grooved, or adhesion/plowing damage develops, the yarn–hook contact resistance and tension-transfer state may change persistently, leading to a sustained deviation of μ from its steady-stage baseline.

The decision target is not “complete failure” in a strict materials sense, but a reproducible online engineering endpoint: when μ[k] rises significantly relative to the steady baseline $\mu_{ss}$ and persists over time, the hook is deemed to have entered an unacceptable accelerated-wear stage. The corresponding onset time is defined as the online wear-life $t_{life}$. A single absolute threshold is not suitable for this problem because the initial friction level may differ among samples, batches, yarn conditions, and tension settings. Therefore, the threshold is referenced to the steady-stage baseline of each test. In addition, isolated spikes may be caused by residual tension ripple, short-term contact disturbance, or communication jitter, and should not be interpreted as wear-life endpoints. For this reason, a persistence-time requirement is introduced together with the relative threshold. The criterion is thus designed to identify a sustained state transition from steady wear to accelerated wear, rather than a single abnormal sample. For comparability across samples/batches and different set tensions, the decision threshold $\mu_{th}$ is first expressed in the original friction-coefficient domain as:

(15) $$\mu_{th}=(1+\delta )\mu_{ss},$$

where δ is the allowable increase of the equivalent friction coefficient relative to the steady baseline. In this study, the nominal threshold increment is set as $\delta_{0}=0.25$ for the main wear-life computation. The value of δ is selected according to the fluctuation level of the steady stage and the required conservativeness of the maintenance decision. Its influence on the final $t_{life}$ output is further evaluated through sensitivity analysis in the results section.

To suppress false triggers from spikes and residual ripple, a persistence-time constraint τ is introduced: the life endpoint is triggered only if μ exceeds $\mu_{th}$ continuously for at least τ [18].

#### 2.4.2. Definition and Extraction of the Steady Baseline $\mu_{ss}$

The steady baseline $\mu_{ss}$ is extracted only from valid samples with q[k]=1, as defined by the data-quality rules in Section 2.3. This prevents invalid samples from contaminating the baseline and shifting the subsequent decision threshold $\mu_{th}$. For each sample and each tension setpoint $T_{set}$, $\mu_{ss}$ is extracted independently, because the steady friction level may vary with the initial contact condition, yarn state, and loading condition.

1. Steady-window and valid-sample set.

With $f_{s}=50\;Hz$ and $T_{s}=0.02\;s$, define a window start index $k_{0}$ and a window length $N_{ss}$ (duration $W_{ss}=N_{ss}T_{s}$). The valid-sample set in the window is given by

(16) $$S[k_{0}]=\{\mu [k]{|k}_{0}\leq k\leq k_{0}+N_{ss}-1,q[k]=1\}$$

Here, $N_{eff}=|S|$. If $N_{eff}$ is insufficient, baseline computation is delayed. In practice, require $N_{eff}/N_{ss}\geq 0.9$; otherwise, slide the window until the condition is satisfied.

2. Steady detection and baseline selection.

In a window meeting the valid-sample ratio, steady detection is based on small fluctuation, bounded local variation, and sufficient persistence. A slight monotonic rise is allowed, since the friction coefficient in the steady-wear stage may still increase gradually with wear progression. For the valid-sample set $S[k_{0}]$ defined in Equation (16), let $\mu_{med}[k_{0}]$ denote the median of the friction-coefficient samples in this set. The robust dispersion of the window is then calculated as:

(17) $$MAD[k_{0}]=median(|\mu -\mu_{med}[k_{0}]|;\mu \in S[k_{0}]),\sigma [k_{0}]=1.4826MAD[k_{0}]$$

If $\sigma [k_{0}]$ is less than $\sigma_{max}$, the window is regarded as steady. In addition, compute the range within the window, $G[k_{0}]=\underset{\mu \in S[k_{0}]}{max}\mu -\underset{\mu \in S[k_{0}]}{min}\mu$, and require that $G[k_{0}]\leq g_{max}$, where $g_{max}=0.02$. To avoid falsely identifying seemingly steady segments, this condition must hold for at least $W_{hold}$. The corresponding steady segment is then represented by the friction-coefficient sample set $\Omega_{stable}$, which is formed by consecutive valid-sample sets $S[k_{0}]$ satisfying the above conditions and covering a duration of at least $W_{hold}$.

(18) $$\mu_{ss}=median(\Omega_{stable})$$

In this experiment, $\sigma_{max}=0.01$ and $W_{hold}=3600\;s$.

#### 2.4.3. Persistent-Exceedance Wear-Life Criterion

The persistent-exceedance criterion is implemented directly in the original equivalent-friction-coefficient domain. According to Equation (15), the decision threshold is defined as $\mu_{th}=(1+\delta )\mu_{ss}$. During criterion computation, only valid samples are used for threshold judgment. If q[k]=1 and $\mu [k]>\mu_{th}$, the current sample contributes to the exceedance counter; otherwise, it is treated as a non-exceedance or interruption sample. Samples with q[k]=0 do not increase the exceedance counter.

Let the persistence time be τ and the corresponding sample count be $N_{\tau }$:

(19) $$N_{\tau }=\lceil \frac{\tau }{T_{s}}\rceil$$

To tolerate brief packet loss or short dips, define a maximum interruption time $\tau_{g}$ with sample count $N_{g}$:

(20) $$N_{g}=\lfloor \frac{\tau_{g}}{T_{s}}\rfloor$$

Use two counters: exceedance counter c[k] and interruption counter g[k], with c[0]=0, g[0]=0. Update rules:

If b[k]=1:

(21) c[k]=c[k−1]+1,g[k]=0.

If b[k]=0:

(22) g[k]=g[k−1]+1.

If $g[k]\leq N_{g}$, the interruption is tolerated and keep c[k]=c[k−1]; if $g[k]>N_{g}$, continuity is broken and reset c[k]=0,g[k]=0.

When $c[k]\geq N_{\tau }$ holds for the first time, trigger life endpoint and define the earliest start of this persistent-exceedance state:

(23) $$k_{life}=k-N_{\tau }+1,t_{life}=k_{life}T_{s}.$$

This definition treats the endpoint as a sustained state rather than a single event, improving robustness to spikes and occasional anomalies.

### 2.5. Experimental Setup and Validation Protocol

#### 2.5.1. Platform Overview

This section validates the full chain “closed-loop tension stabilization → friction coefficient calculation → wear-life output” and links online outputs with offline wear characterization to support consistent comparison in the results section [19]. The experimental design follows three principles: operating-condition consistency, controllability for comparison, and repeatability [20]. Within one life test, line speed, yarn/lubrication state, and contact path are kept constant. Closed-loop constant-tension control stabilizes the average tension near the setpoint, so changes in the friction-coefficient sequence mainly reflect wear evolution rather than drift. Needle hooks with two manufacturer-reported quality grades, namely Grade I and Grade II, are selected for controlled comparison, with repeated tests performed for each grade under the same operating conditions [21].

**Figure 4:**
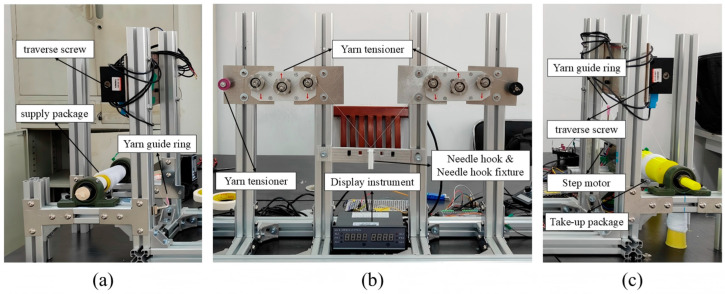
Photographs of the experimental setup: (**a**) yarn supply module; (**b**) tension regulation and needle-hook test module; (**c**) yarn take-up module. Red arrows indicate the yarn contact/wrapping positions on the tension sensors.

To implement the above requirements in a repeatable test platform, the experimental system is organized into three functional modules: yarn supply, tension regulation with needle-hook testing, and yarn take-up. Together, these modules define a stable yarn path, controlled wrap-contact condition, and continuous two-side tension acquisition environment for online friction estimation and wear-life evaluation. The corresponding experimental platform is shown in Figure 4.

**Figure 5:**
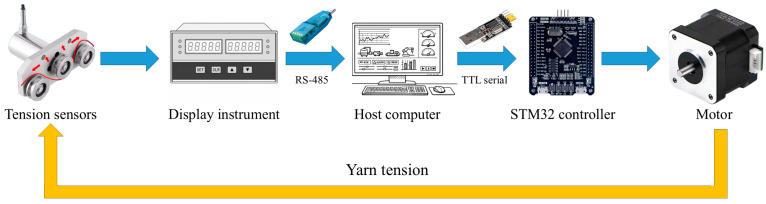
Online monitoring and control workflow. Red arrows indicate the directions of yarn tension at the tension sensors, blue arrows indicate data/control transmission among the modules, and the yellow arrow indicates the yarn-tension feedback path.

Figure 5 summarizes the online monitoring and control workflow, including tension-signal acquisition, data transmission, host-computer processing, STM32-based control, and motor-driven yarn-tension regulation. Tension data measured by the sensors are first read by the display instrument and transmitted to the host computer through RS-485 serial communication. After data processing, the host computer sends control commands to the STM32 controller through transistor-transistor logic (TTL) serial communication, and the controller drives the motor to regulate the yarn tension accordingly. The host-computer data acquisition, online computation, and data-logging functions were implemented using self-developed Python scripts.

Since the algorithmic procedures have been described in Section 2.3 and Section 2.4, this experimental section focuses on the test objects, operating settings, data acquisition, and validation metrics. Data-quality gating, anomaly handling, and noise suppression follow Section 2.3; steady-segment identification, baseline computation, and wear-life criterion follow Section 2.4. Based on these unified rules, the experiments output four comparable elements: closed-loop stability performance, full-time evolution and life output of μ, discrimination across different sample quality grades, and consistency evaluation between offline wear and online outputs in trend and grade-related differences.

#### 2.5.2. Samples and Operating Conditions

Needle hooks with two manufacturer-reported quality grades were selected from samples with the same nominal material and comparable heat-treatment/surface-treatment processes, thereby reducing systematic bias from material and assembly differences. The samples are labeled according to their quality grades, namely Grade I and Grade II. Each sample uses a unified ID scheme. Before installation, appearance and initial-state records are taken, including microscopy images of the tip and working surface and the manufacturer-reported quality grade from datasheets or enterprise maintenance records. During replacement, assembly procedures and yarn path are strictly kept consistent to avoid misinterpreting assembly-induced tension changes as friction-state changes.

**Figure 6:**
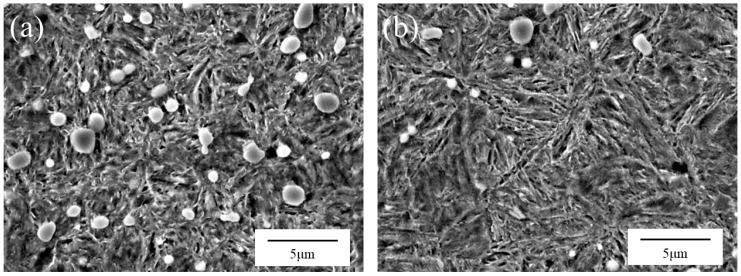
Representative SEM microstructural images of needle hooks with different quality grades before wear testing: (**a**) Grade I; (**b**) Grade II.

The initial material characteristics of Grade I and Grade II needle hooks were further examined before the wear tests to provide material-state evidence for interpreting grade-related performance differences. Previous research on high-carbon steel sheets for knitting needles has shown that alloying and microstructural characteristics can affect hardenability, impact toughness, fatigue life, and service performance of knitting-needle materials [22]. Therefore, the elemental compositions of the two quality grades are summarized in Table 1, and representative pre-test microstructures observed by SEM are further compared in Figure 6.

The elemental-composition results in Table 1 indicate clear differences between the two quality grades, particularly in Mn, P, S, and Cr contents. These differences, together with the SEM microstructural observations shown in Figure 6, provide supplementary evidence that Grade I and Grade II samples differ in their initial material state. This material-state difference provides a basis for interpreting the subsequent online wear-life outputs together with the statistical results and offline wear characterization.

Figure 6 compares the pre-test SEM microstructures of Grade I and Grade II samples, both of which exhibit a martensitic matrix with dispersed bright carbide particles. The bright particle-like phases are mainly carbides, while the matrix is mainly composed of martensitic laths. Compared with Figure 6b, the Grade I sample in Figure 6a shows a finer microstructure, with more abundant and more uniformly dispersed carbide particles and relatively compact martensitic laths. In contrast, the Grade II sample in Figure 6b exhibits a coarser microstructure, with fewer visible carbide particles and larger spacing between martensitic laths. These observations provide supplementary evidence that the two quality grades differ in their initial material state. The finer martensitic structure and more dispersed carbide particles observed in Grade I suggest a possible microstructural basis for its grade-related wear-resistance difference, which is consistent with previous findings that alloying and microstructural characteristics affect the mechanical and fatigue-related performance of high-carbon steel sheets for knitting needles [22]. This observation provides additional material evidence for interpreting the longer online wear-life outputs of Grade I samples reported in the following sections.

Table 2 summarizes the main hardware configuration of the test platform for online tension measurement and wear-life evaluation.

Yarn and lubrication conditions are kept consistent throughout the test [23]. Preferably fix the same yarn batch and record yarn type/material, linear density (count), twist, and oil content or lubrication method. If yarn is replaced, batch number and lubrication changes must be recorded to avoid systematic drift in the friction-coefficient calculation. Yarn path and contact path should remain stable to prevent contact-point drift across different surface regions, which would break comparability across tests [24].

Wrap angle θ is determined by the yarn path and geometry, and should be fixed structurally and calibrated before tests for repeatable clamping. During tests, limiters and anti-slip measures should suppress vibration-induced geometric change so that θ and the contact path remain constant. Speed and tension use a constant-tension strategy: with set tension $T_{set}$, motor speed is adjusted to close the loop, keeping tension slightly fluctuating near the setpoint to reduce masking by operating-condition drift. To prevent yarn breakage due to transients or overshoot, motor speed is saturated by $n_{max}$. In this experiment, $n_{max}=400\;rpm$. When saturation occurs, the output of the incremental PID controller is limited to prevent excessive control action and maintain stable closed-loop operation. Within a single life test, $T_{set}$ and the geometric conditions remain unchanged; speed self-adjusts within $0\sim n_{max}$ under the closed loop. In practice, once the tension converges and the actuator is not persistently saturated, the speed command also converges to a narrow band; thus the yarn line speed is quasi-constant during steady closed-loop operation.

The test duration follows “preset duration first, then compute life”: before starting, set a planned duration $t_{end}$. The system records tension and online computed quantities throughout. After the test, identify the steady segment and compute the baseline $\mu_{ss}$ following Section 2.4.2, then scan the full timeline to obtain $t_{life}$ using the criterion in Section 2.4.3. If no persistent exceedance occurs within $t_{end}$, the sample is recorded as “no failure” and $t_{life}$ is not output. If the test ends early due to protection, it is recorded as an abnormal interruption; if interruption happens after failure, offline computation may still be attempted and the result is marked accordingly.

#### 2.5.3. Data Acquisition and Online Computation

Online measurement uses tension time series as the core input. T1[k] and T2[k] are acquired continuously at a fixed sampling rate with synchronized timestamps to support offline audit. At startup, sensor zero/span checks are performed; then valid data recording begins under steady running. Control-related variables (speed command/actual speed, saturation states, and key limit flags) are synchronously recorded together with the tension signals to support interpretation of fluctuation sources and verification of control-state consistency during comparative tests [25].

Online computation follows “gate first, then compute”: each sample is first checked for validity, and only samples meeting a valid tension range with no obvious packet loss/jumps and no sensor saturation are included in ratio/log computations. At each sample, update R[k] and compute μ[k], while also outputting criterion-related quantities and states for real-time observation. Because derivation is nonlinear and sensitive to relative errors, data-quality control uses a two-level strategy:

1. Real-time gating with constraints on raw tensions and derived variables to eliminate severely distorted points;
2. Offline refinement after steady-segment identification using window statistics (fluctuation degree, jump rate, missing ratio) to further screen samples, ensuring sufficient temporal stability for baseline and life decision.

The goal is to avoid spikes/divergence of R[k] and μ[k] caused by low tension, saturation, or abrupt jumps, so that wear-life output remains insensitive to disturbances and monotonic/interpretable with wear evolution.

All raw tensions, control quantities, computed outputs, and quality flags are written into log files with consistent field names, units, and alignment across tests. Besides μ[k], the system outputs key results such as $\mu_{ss}$, threshold parameters, $t_{life}$ and its trigger time, and anomaly/event marks to support controlled comparison and offline consistency validation.

#### 2.5.4. Controlled Comparison and Repeatability Design

In this study, sample grouping in the experimental section is based on reported quality grades rather than directly on actual service life, because the current dataset is sufficient for comparative discrimination but not yet sufficient to establish a strict quantitative mapping between the online wear-life output $t_{life}$ and actual service life.

To verify discrimination across different quality grades and avoid pseudo-differences caused by operating drift, controlled comparison and repetition are combined. Grade I and Grade II needle hooks are tested under the same yarn path, wrap angle, yarn/lubrication condition, tension-control strategy, and data-quality rules. For each quality grade, repeated samples are tested to evaluate the repeatability of the online wear-life output. If yarn breakage, sensor saturation, or data loss occurs, the corresponding events are marked and processed using the data-validity rules in Section 2.3 to ensure that the computed $t_{life}$ values are based on comparable valid-sample sets.

The evaluation focuses on whether the proposed method can produce stable and interpretable wear-life outputs under controlled comparison conditions. Specifically, $t_{life}$ is expected to show repeatable grade-related differences between Grade I and Grade II samples, while the evolution of μ near the life endpoint should remain consistent with the transition from steady wear to accelerated wear. In addition, the direction of the online wear-life difference should be consistent with offline wear characterization, so that a closed evidence chain can be formed from friction evolution to life output and then to wear morphology.

#### 2.5.5. Offline Characterization and Consistency Verification

Offline characterization provides independent evidence for online outputs. The core goal is to quantify wear under a unified measurement protocol and verify consistency with μ evolution and $t_{life}$. Measurements prioritize pre-test and end-of-test states; if needed, mid-test sampling at representative times can be added to capture staged evidence. Disassembly/reassembly must be consistent, and measurement position, imaging area, magnification, and illumination should be fixed to avoid geometry bias or misinterpretation from changing observation conditions.

Indicators follow a “few but precise” principle: two quantitative indicators plus image evidence. Quantitative indicator 1 is geometric wear (e.g., tip wear-scar width). Quantitative indicator 2 is an auxiliary measure reflecting contact wear (e.g., key cross-section size change, worn-area size), chosen once and kept consistent. Image evidence (microscopy/SEM) identifies typical morphologies (abrasive wear, adhesion/plowing) and links them to stages of friction evolution. If multiple indicators are available, use the main indicator as the reference and others as supporting evidence to avoid diluted statistics or conflicting explanations.

Consistency verification includes trend consistency and grade-related consistency. Trend consistency requires offline wear to evolve in a direction matching online μ, and to show intensified wear evidence near the end of life. Grade-related consistency requires that offline wear differences align with $t_{life}$ differences: samples from the grade with shorter $t_{life}$ should show heavier wear or more severe morphology. Samples that violate consistency should be traced back using quality flags and event logs, checking for non-wear factors such as yarn breakage, contact-path drift, saturation, or stops; such samples should be separately marked or excluded in summaries.

## 3. Results and Analysis

### 3.1. Evolution of μ(t) and Wear-Life Outputs

This section reports full-time evolution curves of tension and friction indicators for representative samples, and shows the offline computation of $\mu_{ss}$ and $t_{life}$ following Section 2.4. All results are based on valid samples with q[k]=1; steady-segment identification, baseline computation, and criterion parameters follow Section 2.4 and are not repeated here.

Table 3 summarizes the individual online wear-life results, providing a quantitative basis for comparing different samples, quality grades, and tension setpoints.

For each $T_{set}$, $\mu_{ss}$ was extracted independently for each sample, and $t_{life}$ was calculated using the same baseline-referenced persistent-exceedance criterion.

Based on the individual results in Table 3, the statistical summaries for each quality grade and tension setpoint are further calculated and listed in Table 4.

The statistical results in Table 4 indicate that Grade I samples exhibited longer mean online wear-life than Grade II samples under all tested tension setpoints. Although the absolute $t_{life}$ varied with $T_{set}$, the grade ranking remained consistent, indicating that the proposed baseline-referenced criterion maintains relative discrimination capability under different controlled loading conditions.

To examine the influence of the threshold parameter δ on the wear-life output, a sensitivity analysis was performed by recalculating $t_{life}$ under different δ settings. The nominal threshold increment used in the main analysis was $\delta_{0}=0.25$, and two neighboring settings, $0.9\delta_{0}$ and $1.1\delta_{0}$, were further tested. The purpose of this analysis is not to determine a unique optimal threshold, but to evaluate whether the grade-ranking result remains stable under moderate threshold variation.

The sensitivity results in Table 5 show that decreasing δ led to earlier $t_{life}$ detection, whereas increasing δ delayed the detected endpoint. However, within the tested range of δ, Grade I consistently exhibited longer average $t_{life}$ than Grade II. This result indicates that the quality-grade discrimination is not sensitive to moderate variation of the threshold parameter.

**Figure 7:**
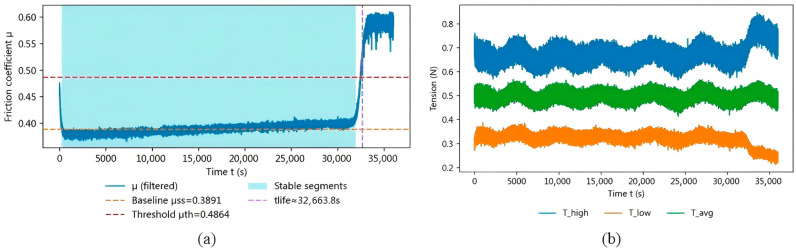
Representative online monitoring results for a typical sample: (**a**) time evolution of the equivalent friction coefficient μ with the baseline, threshold, stable segment, and detected $t_{life}$; (**b**) full-time-domain tension signals.

The representative full-time-domain results indicate that the online friction indicator exhibits a clear stage-wise evolution, while the closed-loop regulation keeps the operating tension within a comparatively stable range throughout the test. As shown in Figure 7a, the equivalent friction coefficient passes through a run-in stage, a relatively stable stage, and a subsequent accelerated-rise stage. Meanwhile, Figure 7b shows that the average tension remains within a relatively stable range under closed-loop regulation. Together, these results support that the proposed closed-loop strategy provides the operating-condition consistency required for reliable online friction derivation.

**Figure 8:**
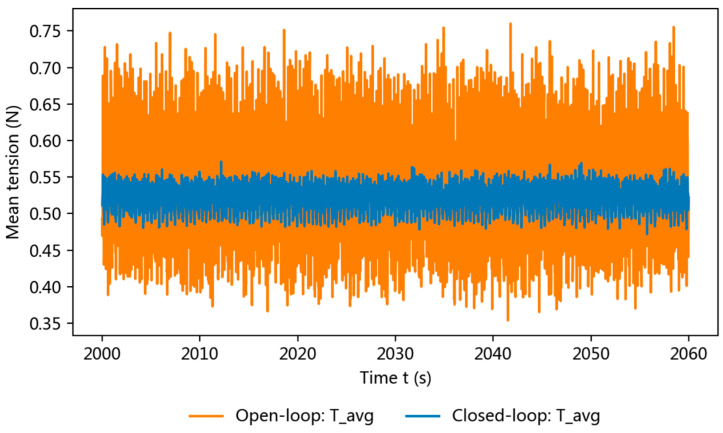
Comparison of tension responses under open-loop and closed-loop conditions.

Figure 8 compares the tension responses under open-loop and closed-loop conditions to evaluate the effect of the proposed closed-loop strategy on operating-condition stability. Compared with open-loop operation, the closed-loop controller suppresses long-term drift and reduces fluctuation around the setpoint, thereby improving the stability and comparability of the signals used for subsequent friction derivation and wear-life evaluation.

**Figure 9:**
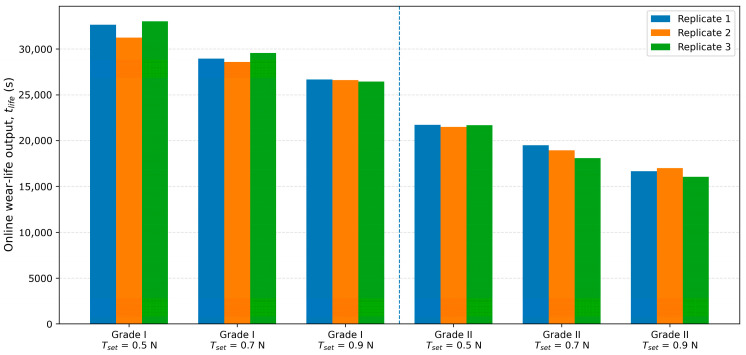
Relationship between sample quality grade and online wear-life output.

Figure 9 presents the relationship between sample quality grade and the computed online wear-life output, providing a direct visual comparison of the grade-discrimination capability. The plot provides a direct visual comparison between the reported quality grade and the online evaluation result across different samples and quality grades.

Taken together, the results in Figure 7, Figure 8 and Figure 9 show that the proposed closed-loop strategy effectively suppresses long-term operating-condition drift and keeps the tension input comparatively stable during the test. Under this condition, the derived friction indicator exhibits a clear stage-wise evolution, and the wear-life criterion yields a distinguishable online wear-life output once persistent exceedance occurs. Moreover, Grade I and Grade II samples show correspondingly different online wear-life outputs, indicating that the proposed method can distinguish samples of different quality grades with good reproducibility.

### 3.2. Consistency with Offline Characterization

This section verifies consistency between online wear-life $t_{life}$ and offline wear characterization. The offline measurement protocol follows Section 2.5.5, while $\mu_{ss}$ and $t_{life}$ computation follows Section 2.4.

To provide independent evidence of the sample wear state and to verify the consistency of the online wear-life output with the observed geometric and morphological changes, the offline characterization results are summarized in Table 6.

**Figure 10:**
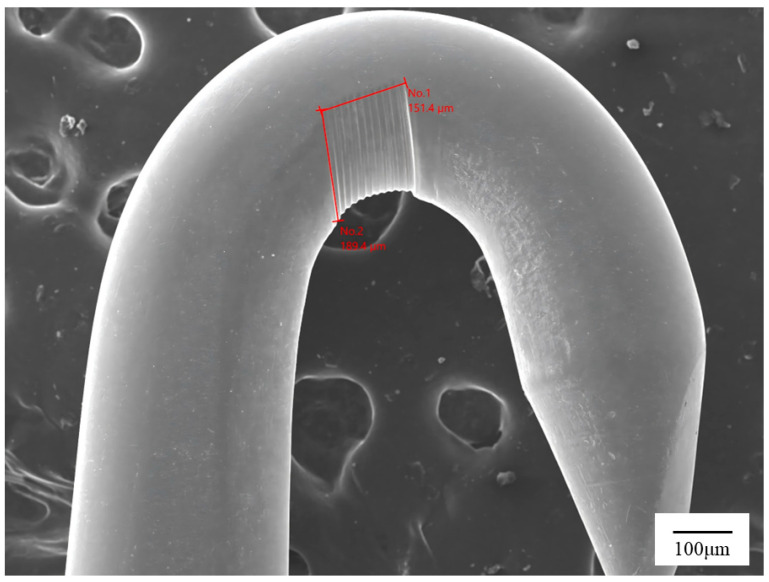
Representative scanning electron microscopy (SEM) images of wear marks on the needle-hook surface.

To further examine the microscopic features associated with the wear process inferred online, representative SEM images of the needle-hook surface are presented in Figure 10. These images provide morphological evidence of progressive surface damage and serve as a microstructural complement to the offline geometric measurements summarized in Table 6.

Combined with the quantitative results in Table 6, the SEM observations confirm that observable wear develops on the tip and working surface after testing, and that the severity of wear differs between Grade I and Grade II samples in a direction consistent with their reported quality grades. In addition, samples with heavier offline wear tend to reach persistent exceedance earlier and thus yield smaller online wear-life outputs. This agreement between offline wear evidence and online friction/life results supports the consistency, interpretability, and life-level discrimination capability of the proposed method.

## 4. Discussion

**Figure 11:**
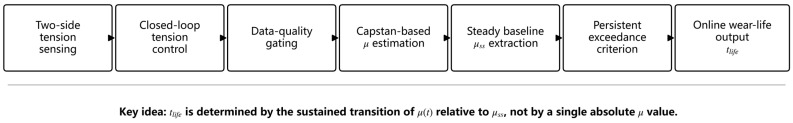
Innovation flow of the proposed online wear-life evaluation method. Arrows indicate the sequential data-processing and decision-making flow.

Before discussing the reliability, grade-discrimination capability, and engineering boundary of the proposed method, the methodological innovation flow is summarized in Figure 11. The core idea is to convert two-side yarn-tension signals into a baseline-referenced online wear-life output through closed-loop tension control, data-quality gating, capstan-based equivalent-friction estimation, steady-baseline extraction, and persistent-exceedance judgment.

### 4.1. Reliability of the Online Wear-Life Output

The reliability of the proposed method comes from converting raw two-side tension signals into a controlled, screened, and baseline-referenced life output. In this study, the equivalent friction coefficient μ is not used as a single absolute life index, because the steady friction level may vary among samples due to initial contact state, material state, yarn condition, and loading condition. Therefore, the life judgment is based on the sustained transition of μ(t) relative to its own steady baseline $\mu_{ss}$, rather than on the instantaneous value of μ.

This design improves robustness in two respects. First, closed-loop average-tension control reduces operating-condition drift, so that the long-term change in μ(t) is more likely to reflect yarn–hook contact evolution rather than uncontrolled tension variation. Second, data-quality gating prevents low-tension samples, abnormal ratios, missing data, and invalid samples from entering μ computation, baseline extraction, and persistent-exceedance counting. As a result, the calculated $t_{life}$ is less affected by occasional signal disturbance and is more closely related to a sustained change in yarn–hook tension-transfer behavior.

### 4.2. Grade Discrimination Based on $t_{life}$

The experimental results show that the proposed $t_{life}$ output can distinguish Grade I and Grade II needle hooks under the tested controlled conditions. This discrimination does not rely on a fixed absolute friction-coefficient value, but on the time position at which the μ(t) sequence begins to deviate persistently from its own steady baseline. Therefore, even when the steady friction levels of different samples are close or partially overlapping, the method can still compare their relative wear-life behavior through $t_{life}$.

The consistency between online $t_{life}$ outputs and offline wear characterization further supports the physical interpretability of the method. A key advantage of the proposed algorithm is that it converts the friction-coefficient evolution into an interpretable wear-related endpoint. Samples that reach the persistent-exceedance state earlier generally show more severe geometric wear and surface damage after testing. This indicates that the baseline-referenced transition in μ(t) is associated with the actual development of wear on the needle-hook surface. Therefore, $t_{life}$ can serve as an interpretable online indicator linking friction evolution with wear progression, rather than a purely empirical time output. This agreement suggests that short-duration online measurements can provide useful information for evaluating relative needle-hook wear-life levels under controlled operating conditions.

### 4.3. Robustness and Engineering Boundary

The threshold-sensitivity analysis indicates that moderate variation of the threshold increment changes the detected $t_{life}$ slightly but does not change the grade-related ranking. This means that the proposed criterion is not determined by a single accidental threshold choice, but reflects a relatively stable transition feature in the friction-evolution sequence. Therefore, the method shows a certain degree of robustness for controlled comparative tests.

The present $t_{life}$ should still be regarded as an online engineering endpoint rather than a direct month-level field service-life prediction. Its applicability depends on stable wrap angle, stable contact path, consistent yarn and lubrication conditions, reliable two-channel tension acquisition, and sufficient valid samples. Establishing a quantitative mapping between $t_{life}$ and actual replacement intervals will require larger datasets, different process conditions, and long-term maintenance records.

## 5. Conclusions

This paper proposes a deployable method for online friction measurement and wear-life determination of textile needle hooks. Under constant-tension closed-loop control, an equivalent friction coefficient is derived from the slack-side and tight-side tension signals based on the capstan relation. Data-quality control is ensured through tension gating, ratio clipping, missing-sample handling, and outlier rejection. On this basis, a baseline-referenced wear-life criterion is established using a “relative threshold + persistence time” approach, which identifies the life endpoint as the onset of a state of sustained exceedance rather than a transient spike-sensitive event.

The experimental results demonstrate that the closed-loop strategy improves tension stability and operating-condition consistency, enabling the derived friction indicator to exhibit a clear stage-wise evolution. The proposed criterion yields distinguishable online wear-life assessments for samples of varying manufacturer-reported quality grades, with trends consistent with offline microscopy and SEM wear evidence. These findings support the interpretability, reproducibility, and engineering applicability of the method for maintenance-oriented life evaluation of textile needle hooks.

The method is applicable under conditions where the wrap angle and contact path remain stable, the yarn batch and lubrication state are consistent, and the two tension channels are reliably synchronized. Future work can further improve robustness by strengthening the structural constraint of the yarn path, enhancing dual-channel anti-noise and synchronization performance, and developing adaptive parameter-selection and correction strategies for different needle types and process conditions.

## Figures and Tables

**Table 1:** Elemental composition of needle hooks with different quality grades.

Grade	C (%)	Si (%)	Mn (%)	P (%)	S (%)	Cr (%)
I	0.980	0.220	0.510	0.012	0.002	0.330
II	0.970	0.220	0.460	0.014	0.003	0.260

**Table 2:** Main hardware configuration of the experimental platform.

Device	Model	Manufacturer
Stepper motor	57	Changzhou Xingbo Motor Co., Ltd., Changzhou, China
Stepper motor driver	DM542	Changzhou Xingbo Motor Co., Ltd., Changzhou, China
Tension sensor	ZNZL-5N	Bengbu Zhongnuo Sensor Co., Ltd., Bengbu, China
Dual-channel tension display meter	ZN-S	Bengbu Zhongnuo Sensor Co., Ltd., Bengbu, China
Microcontroller unit (MCU)	STM32F103RCT6	STMicroelectronics N.V., Geneva, Switzerland

**Table 3:** Online wear-life results for individual samples under different tension setpoints.

Sample ID	Quality Grade	$T_{set}$ (N)	$t_{end}$ (h)	$t_{life}$ (s)	$\mu_{ss}$
I-05-01	Grade I	0.5	10	32,663.8	0.3891
I-05-02	Grade I			31,228.3	0.3076
I-05-03	Grade I			33,028.6	0.2780
II-05-01	Grade II			21,734.2	0.3957
II-05-02	Grade II			21,506.6	0.3961
II-05-03	Grade II			21,689.8	0.2591
I-07-01	Grade I	0.7		28,958.2	0.2580
I-07-02	Grade I			28,604.2	0.2494
I-07-03	Grade I			29,569.1	0.2517
II-07-01	Grade II			19,488.6	0.2412
II-07-02	Grade II			18,933.5	0.2399
II-07-03	Grade II			18,099.5	0.2536
I-09-01	Grade I	0.9		26,674.2	0.2546
I-09-02	Grade I			26,611.1	0.2504
I-09-03	Grade I			26,453.1	0.2466
II-09-01	Grade II			16,683.9	0.2556
II-09-02	Grade II			17,004.2	0.2553
II-09-03	Grade II			16,043.2	0.2580

**Table 4:** Statistical summary of online wear-life outputs under different tension setpoints.

$T_{set}$ (N)	Quality Grade	$t_{life\_avg}$ (s)	SD (s)	CV (%)	Grade Ranking
0.5	Grade I	32,306.9	951.7	2.95	I > II
0.5	Grade II	21,643.5	120.6	0.56	
0.7	Grade I	29,043.8	488.1	1.68	I > II
0.7	Grade II	18,840.5	699.2	3.71	
0.9	Grade I	26,579.5	113.9	0.43	I > II
0.9	Grade II	16,577.1	489.3	2.95	

**Table 5:** Sensitivity analysis of online wear-life outputs under different δ settings.

$T_{set}$ (N)	δ	Quality Grade	$t_{life\_avg}$ (s)	$\mathrm{Change}\;\mathrm{Relative}\;\mathrm{to}\;\delta_{0}$ (%)	Grade Ranking
0.5	$0.9\delta_{0}$	Grade I	32,222.4	−0.26	I > II
	$0.9\delta_{0}$	Grade II	21,592.0	−0.24	
	$\delta_{0}$	Grade I	32,306.9	0	I > II
	$\delta_{0}$	Grade II	21,643.5	0	
	$1.1\delta_{0}$	Grade I	32,328.2	0.07	I > II
	$1.1\delta_{0}$	Grade II	21,845.5	0.93	

**Table 6:** Offline characterization results of tested needle hooks under the baseline tension condition.

Sample ID	Quality Grade	$T_{set}$ (N)	$t_{life}$ (s)	$\Delta w_{1}$ (μm)	$\Delta w_{21}$ (μm)	$\Delta w_{22}$ (μm)	$\Delta w_{23}$ (μm)	$\Delta w_{3}$ (μm)
I-05-01	Grade I	0.5	32,663.8	36.8	86.1	86.6	87.2	160.4
I-05-02	Grade I		31,228.3	40.2	87.3	87.6	88.1	167.1
I-05-03	Grade I		33,028.6	37.7	84.2	85.3	85.5	151.4
II-05-01	Grade II		21,734.2	42.1	85.2	85.6	85.9	176.5
II-05-02	Grade II		21,506.6	40.4	84.4	84.8	85.5	171.2
II-05-03	Grade II		21,689.8	43.4	87.5	88.1	88.4	186.3

## Data Availability

The data presented in this study are available on request from the corresponding authors. Public access to the full dataset is restricted because some materials and supporting records contain confidential technical information related to the experimental platform, including aspects of the mechanical structure and specific operating parameters, which may be used in follow-up research. The study reported in this manuscript has been completed, and the data supporting the reported findings are valid. Raw data and the minimal dataset necessary to evaluate the main findings can be provided to the Editorial Office and reviewers upon request for confidential assessment.
